# A rare clinical presentation of third part duodenal perforation due to post‐endoscopic retrograde cholangiopancreatography stent migration on advanced stage peri‐ampullary tumor

**DOI:** 10.1002/jgh3.12608

**Published:** 2021-07-02

**Authors:** Budhi Ida Bagus

**Affiliations:** ^1^ Department of Surgery Sebelas Maret University Surakarta Indonesia

**Keywords:** duodenal perforation, endoscopic retrograde cholangiopancreatography, peri‐ampullary tumor, stent migration

## Abstract

As a diagnostic and therapeutic treatment role on malignant biliary obstruction, endoscopic retrograde cholangiopancreatography (ERCP) has already been used as a routine procedure, especially for palliative treatment on advanced stage peri‐ampullary tumor. This minimal invasive procedure has many early or late complications such as bleeding, post‐ERCP pancreatitis, perforation, cholangitis, and the rare duodenal perforation from the stent migration. The current review reported the incidence of stent erosion associated with duodenal perforation was only 1% for this palliative procedure. We report a 75 years old male patient with diffuse abdominal tenderness 7 days after palliative ERCP stent placement for malignant biliary obstruction, metal stent could not be placed, and plastic stent placement had been done. There was no post‐ERCP pancreatitis found during the first 24 h. The patient came to the emergency with clinical sign and symptoms of diffuse peritonitis; abdominal X‐ray found no free intraperitoneal air. Exploratory laparotomy was performed, and we found bile leak from the third part of perforated duodenal with 5 mm in diameter, plastic stent exposed from the perforation site, and no active bleeding. We performed primary suture of the duodenum, cholecysto‐enteric bypass, pyloric exclusion, gastro‐jejunostomy bypass, and braun anastomosis. Jejunostomy feeding has been placed. There were no postoperative cardiopulmonary complication, and the patient could tolerate well for oral intake and discharged from hospital at 10th postoperative day (POD). This rare duodenal perforation complication could happen even in plastic stent placement during the ERCP procedure, and early management was needed to gain the favorable outcome.

## Graphical abstract

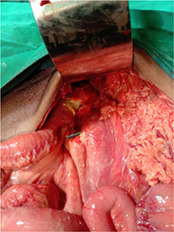

Duodenal perforation due to plastic stent migration during endoscopic retrograde cholangiopancreatography for malignant biliary obstruction was an uncommon complication. Although this was a rare complication, it could still happen even in experienced endoscopist. Better education to the patient plays an important role in early diagnosis of this case to achieve a better clinical outcome.

## Introduction

Since it has been announced, performed safely on biliary drainage procedure, and first published by Soehendra N in 1980, endoscopic retrograde cholangiopancreatography (ERCP) is a routine procedure for diagnostic and therapeutic treatment on benign and malignant biliary obstruction.^1^ Bile duct stones were the most common indication to perform this kind of procedure on benign obstruction. On the other hand, in malignant obstruction cases, ERCP could be the preferred treatment option for biliary drainage as a palliative treatment for the advanced stage periampullary tumor.^1^, ^2^

ERCP and stent placement, especially covered or uncovered self‐expandable metal stent (SEMS), is the best option to treat malignant biliary obstruction, but in some condition, plastic stent could be used as an alternative.^1^, ^3^

This minimally invasive procedure has many early and late complications such as bleeding, post‐ERCP pancreatitis, perforation, cholangitis, and the rare duodenal perforation from the stent migration. The incidence of stent erosion associated with duodenal perforation was only 1% following this palliative procedure.^3^, ^4^

## Case Report

**Figure 1:**
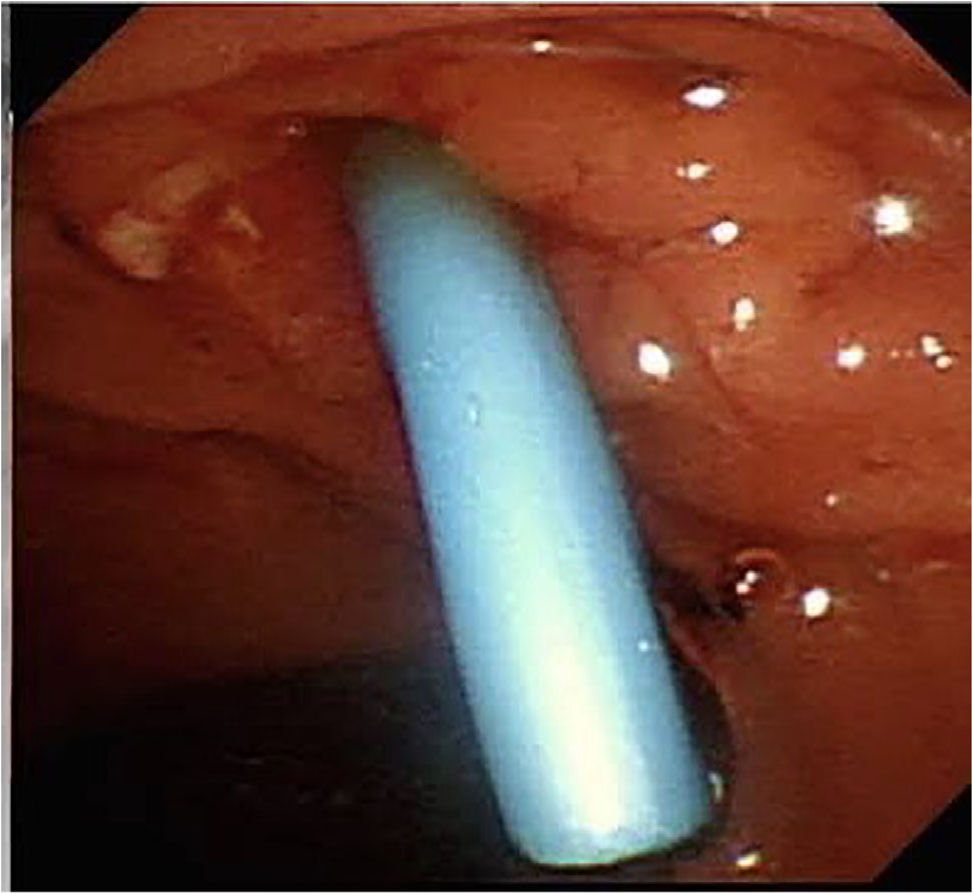
Plastic stent placement.

**Figure 2:**
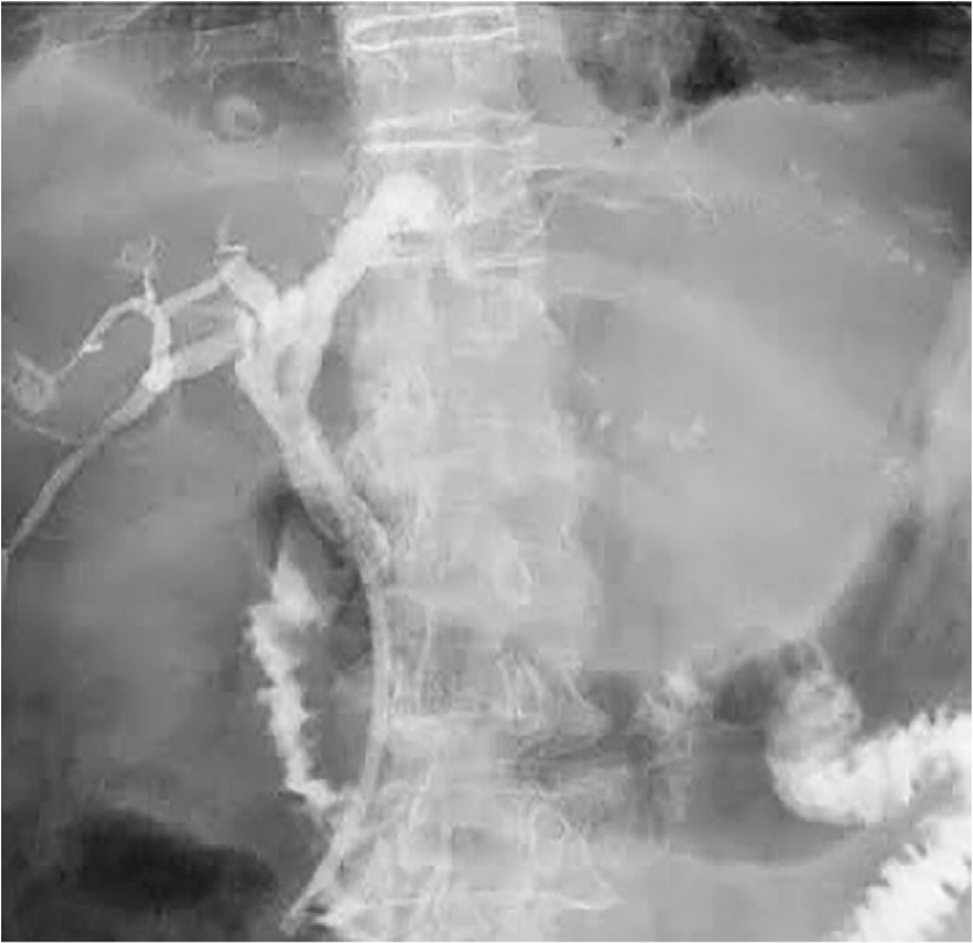
Fluoroscopy view.

**Figure 3:**
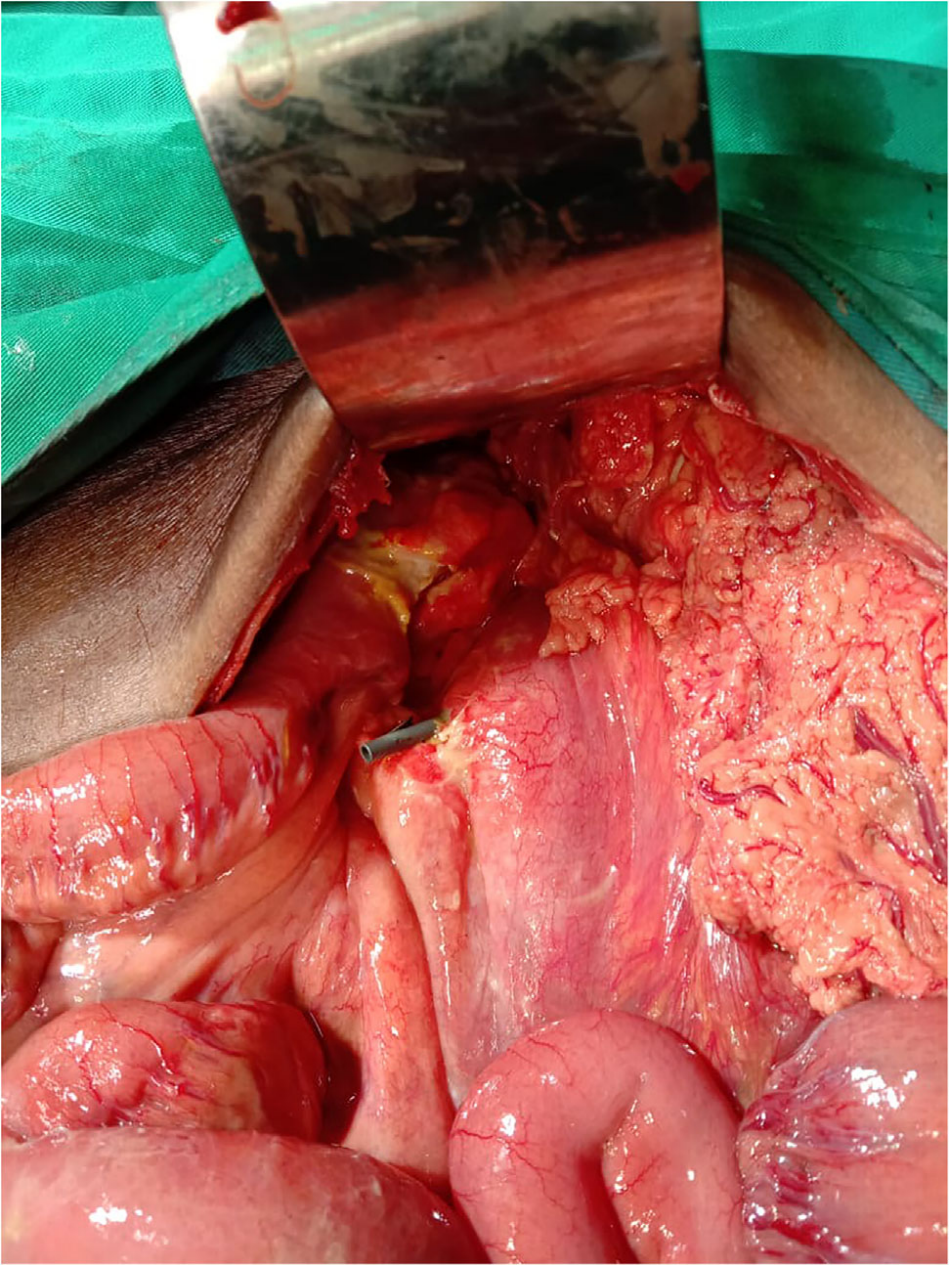
Perforated third part of duodenum with stent migration.

A 75‐year‐old male patient came to the emergency department with diffuse abdominal tenderness 7 days after palliative ERCP stent placement for malignant biliary obstruction and cholangitis; the metal stent could not be placed because of the distal CBD stenosis and unsuccessful SEMS placement, so plastic stent placement had been done (Figs 1, 2). There were no immediate complications within 24 h after the procedure and discharged well after 3 days. The patient came to the emergency department with clinical signs and symptoms of diffuse peritonitis, and the abdominal X‐ray found no free intraperitoneal air. Upon exploratory laparotomy, a perforation site was found at the third part of the duodenum, measuring 5 mm in diameter with plastic stent exposing out from the perforation site (as shown in Fig. 3). The distal tip of the plastic stent migrated to the third part of the duodenum and perforated at this site. We performed primary suture of the duodenum, cholecysto‐enteric bypass, pyloric exclusion, gastro‐jejunostomy bypass, and braun anastomosis. Jejunostomy feeding has been placed at the end of the procedures. There were no postoperative cardiopulmonary complication and the patient could tolerate well for oral intake and discharged from hospital at 10th postoperative day. This case study has already been approved by Health Research Ethic Committee of Moewardi General Hospital, Indonesia. Ethical clearance number: 345/III/HREC/2021.

## Discussion

ERCP is a minimally invasive procedure in diagnostic and therapeutic option of biliary obstruction cases, not only in benign biliary obstruction but also for malignant biliary obstruction as a palliative biliary drainage. When performing the ERCP, we must pay attention on the difficulties with anatomical variation and side‐viewing scope. Bleeding and intestinal injuries related to instrumentation are the most common intra‐procedural complications, which could happen even with experienced endoscopists. When these complications occur during the procedure, it can be managed directly either using over or through the scope clip.^5^

Duodenal perforation during ERCP procedure is an absolutely rare complication, a review by Cirocchi et al. in 2016 described the incidence of duodenal perforation after ERCP range from 0.09 to 1.67%, and the mortality was reported up to 8% in high‐volume referral center.^5^, ^6^ The keypoint in the management of duodenal perforation related to ERCP is early diagnosis; early diagnosis and management lead to favorable clinical outcomes with no postoperative morbidity and lower mortality rate.^7^ But these kinds of complications sometimes have been diagnosed lately and delayed presentation following with intraperitoneal contamination, even with septic condition.^8^ In this case, emergency surgical treatment was the appropriate option. Nonoperative management (NOM) was not the preferred option if the patient present with clinical sign and symptoms of peritonitis and already in septic condition. In stable, localized retroperitoneal perforation with minimal intra‐abdominal contamination, this NOM could achieve better clinical outcome.^8^, ^9^, ^10^

In this case report, presented with a late complication 7 days after ERCP for malignant biliary obstruction, so the preferred treatment was emergency exploratory laparotomy. If this complication has been found during ERCP procedure, direct clipping following the procedure was the recommended option.

The last procedure (jejunal feeding) might be the remaining controversial treatment option in critically ill patients. Some studies do not recommend postoperative feeding using an intraoperative tube placement as it may increase postoperative morbidity. However, in this case, in view of prolonged period of intra‐abdominal bile contamination, there may be a risk of developing postoperative paralytic ileus. Therefore, by placing a jejunal feeding tube, early resumption of intestinal feeding would result in better clinical outcome.^7^, ^8^

Duodenum is the most common site of stent migration, which leads to perforation, and this migration could happen in up to 6% of stent placement during ERCP for biliary drainage procedures; although the stent migration that could lead to perforation can be found in less than 1% of cases, this is extremely rare complication related to ERCP.^9^ As described before, an early diagnosed case and in stable patients, it could be managed by through‐the‐scope clip and stent evacuation at the same time of procedure.^8^

Many factors play an important role on distal migration of the plastic stent after ERCP. A multivariate analysis study reported by Xiang‐lei et al. in 2020 described the risk factors such as benign biliary stricture (BBS), a proximal stricture, the length of stent more than 10 cm, length of the proximal stent above the proximal end of the stricture (>2 cm), and duration of stent retention for more than 3 months. The results of logistic regression analysis of this study reported only BBS, length of the stent, and duration of stent retention was statistically relevant.^9^, ^10^ According to this risk factor study, the length of the stent in this case was more than 10 cm, which was associated with stent migration and leading to duodenal perforation. In another study, Sanchez‐Tembleque et al. suggested the flexibility of the stent could impact the possibility of stent migration and duodenum injury. The inflexibility of these plastic stent has higher possibility and influence on this complication.

## Conclusion

Duodenal perforation caused by plastic stent placement after ERCP is a rare complication. Early diagnosis and subsequent management are needed to achieve favorable outcomes in such cases.

### Informed consent

The patient has already been informed about the purpose of this case study and obtained patient permission on using of the clinical material such as clinical finding during operation for the publication.
